# Supplementary material for: Bombyx mori Pupae Efficiently Produce Recombinant AAV2/HBoV1 Vectors with a Bombyx mori Nuclear Polyhedrosis Virus Expression System
Source: Viruses. 2021 Apr 18;13(4):704. doi: 10.3390/v13040704 (PMC8073075; doi:10.3390/v13040704)
Supplement: Supplementary file 1 [file viruses-13-00704-s001.zip › viruses-1168268-supplementary.pdf]

Supplementary materials

Bombyx mori Pupae Efficiently Produce Recombinant AAV2/HBoV1 Vectors with a *Bombyx mori* Nuclear Polyhedrosis Virus Expression System

Qian Yu[1], Pengfei Chang[1], Xiaoxuan Liu[1], Peng Lü[1], Qi Tang[1], Zhongjian Guo[1],

Jianming Qiu[2], Keping Chen[1*] and Qin Yao[1*]

1 School of Life Sciences, Jiangsu University, Zhenjiang 212013, China; qianyu@ujs.edu.cn (Q.Y.); 15751000531@163.com (P.C.); 838435143@qq.com (X.L.); penglu@ujs.edu.cn (P.L.); tangqi1224@163.com; (Q.T.); gzh762677@ujs.edu.cn (Z.G.)

2 Department of Microbiology, Molecular Genetics and Immunology, University of Kansas Medical Ceter, Kansas City, KS 66160, USA; jqiu@kumc.edu

* Correspondence: kpchen@ujs.edu.cn (K.C.); yaoqin@ujs.edu.cn (Q.Y.); Tel.: +86-051188791923 (K.C.); +86-13952940568 (Q.Y.)

Method S1: Plasmid construction

The two recombinant plasmids used in the project were constructed into pFastBacDual cloning vector (Invitrogen, Carlsbad, CA, USA) which was described in the previous work [1].

pFastBacAAV2ITR-eGFP: This BEV transfer plasmid was constructed by inserting a 5.4 kb AAV2 inverted terminal repeat (ITR)-flanked proviral DNA into the vector. For the convenience of subsequent experiments, an open reading frame (ORF) of an enhanced green fluorescent protein controlled by the P10 promoter was included between the ITRs.

pFastBac-AAV2Rep-HBoV1Cap: In another BEV transfer plasmid, we first synthesized a 637-bp DNA fragment containing a partially codon-optimized (opt) Rep78 ORF [2], and amplified the full-length optRep78/52 ORF using overlapped PCR, and this resulted in pFastBacDual-AAV2Rep. Secondly, we also synthesized a 390-bp DNA fragment containing an optimized HBoV1 sequence between VP1 AUG and VP3 AUG and amplified the full length optVP1/2/3 ORF using overlapped PCR, which was then cloned into the pFastBacDual-AAV2Rep to obtain the pFastBac-AAV2Rep-HBoV1Cap.

Method S2: rAAV2/HBoV1 purification protocol.

(1) After four freezing–thawing cycles, the cells were sonicated at 70% power for 3 min (1 min periods of sonication with 1 min intervals).
(2) The samples were treated with DNase I at 37 °C for 45 min and centrifuged at 8,000 rpm for 20 min.
(3) Then, the clarified solutions were filtered with 0.45 μm filters (Millipore Millex, Tullagreen, Carrigtwohill, Co. Cork, IRL), and we obtained the crude virus samples.
(4) The rAAV was purified by means of ultracentrifugation using two centrifugation media, including cesium chloride (CsCl) gradient and iodixanol gradient.
(5) For the CsCl gradient ultracentrifugation, CsCl was added into the crude cell lysate to adjust it to a final density of about 1.40 g/mL [3]. The samples were loaded into centrifugation tubes (Beckman Coulter, Kraemer Blvd. Brea, CA, USA) and centrifuged at 41,000 rpm for 36 h at 20 °C using an SW41Ti rotor in an Optima™XPN ultracentrifuge (Beckman Coulter, Kraemer Blvd. Brea, CA, USA).
(6) For the iodixanol gradient ultracentrifugation, we formed an iodixanol step gradient as previously reported [4,5]. Underlying the viral fluid, we added 3 mL of 15% iodixanol solution directly into the bottom of the tube. In the same way, 2 mL of 25% iodixanol solution, followed by 1 mL of 40% iodixanol and 1 mL of 54% iodixanol gradient solution, was added into the bottom of the tube. Then the samples were centrifuged at 32,000 rpm for 5 h at 20 °C using an SW41Ti rotor in an Optima™XPN ultracentrifuge (Beckman Coulter, Kraemer Blvd. Brea, CA, USA).
(7) After the centrifugation, the samples in the centrifuge tubes were carefully collected from top to bottom in a volume of 0.5–1 mL.
(8) Next, we verified the rAAV2/HBoV1 vector by SDS-PAGE combined with Western blotting.
(9) The components containing virions were collected and dialyzed against PBS overnight using a 10 kDa dialysis membrane (Sangon, Shanghai, China) to remove the media.
(10) Then, we measured the vector titer and the purity of the dialyzed fraction by qPCR and SDS-PAGE combined with Western blotting.
(11) Finally, we concentrated the vectors using Amicon Ultra-15 centrifugal filter units (MWCO, 100 kDa; Merck Millipore) and stored the samples at 80 °C.

Result S1: Western Blotting of infected larva and pupa

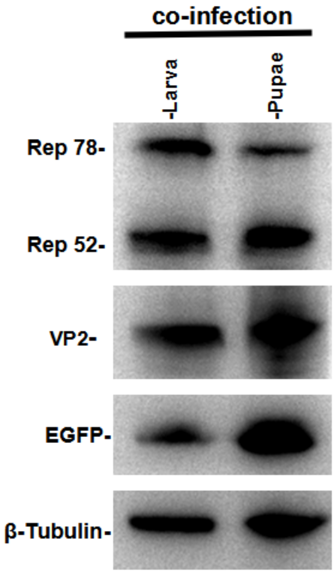

Figure S1. Western Blotting of infected larva and pupa. After 96h of co-infection with recombinant baculovirus, a silkworm larva or a pupa which had expressed eGFP was frozen and ground to prepare protein samples. Western blotting was used to verify the expression of AAV2 Rep 78/52, HBoV1 VP2 and eGFP. A monoclonal antibody against β-tubulin (Proteintech, Wuhan, Hubei, China) was used at a 1:2000 dilution to normalize the added protein content.

Primer sequences:

Table S1: The primers used in the experiment

Primers	Sequences(5'→3')
qPCR-F	CAC AAC ATC GAG GAC GGC AG
qPCR-R	CTT GTA GAG CTC GTC CAT GCC G
AAV2 rep-F	GTACATGGAGCTGGTCGGGTG
AAV2 rep-R	ATTGTTCAAAGATGCAGTCATCC
HBoV1 cap-F	CAACACTTTATTGATGTTTG
HBoV1 cap-R	GCGACACTGACATTCAAGAC
eGFP-F	GTTCAGGGGGAGGTGTGGGAG
eGFP-R	GTTCAGGGGGAGGTGTGGGAG
Bacmid-AAV2ITR-eGFP-F	GCTATAGTTCTAGTGGTTGGCTACG
Bacmid-AAV2ITR-eGFP-R	CCGGATGAAGTGGTTCGCATC
Bacmid-AAV2rep-HBoV1cap-F	CGCGGCGTTGTGACAATTTACC
Bacmid-AAV2rep-HBoV1cap-R	GTTCAGGGGGAGGTGTGGGAG
M13-F	CCCAGTCACGACGTTGTAAAACG
M13-R	AGCGGATAACAATTTCACACAGG

References

1. Deng, X.; Zou, W.; Yan, Z.; Qiu, J. Establishment of a recombinant aav2/hbov1 vector production system in insect cells. *Genes* **2020**, *11*, 439.
2. Smith, R.H.; Levy, J.R.; Kotin, R.M. A simplified baculovirus-AAV expression vector system coupled with one-step affinity purification yields high-titer rAAV stocks from insect cells. Mol. Ther. 2009, 17, 1888–1896.
3. Kimura, T.; Ferran, B.; Tsukahara, Y.; Shang, Q.; Desai, S.; Fedoce, A.; Pimentel, D.R.; Luptak, I.; Adachi, T.; Ido, Y.; et al. Production of adeno-associated virus vectors for in vitro and in vivo applications. *Sci. Rep.* **2019**, *9*, 13601.
4. Meyer, N.; Davulcu, O.; Xie, Q.; Silveria, M.; Zane, G.M.; Large, E.; Chapman, M.S. Expression and Purification of Adeno-associated Virus Virus-like Particles in a Baculovirus System and AAVR Ectodomain Constructs in E. coli. *Bio Protoc.* **2020**, *10*, e3513.
5. Fripont, S.; Marneffe, C.; Marino, M.; Rincon, M.Y.; Holt, M.G. Production, Purification, and Quality Control for Adeno-associated Virus-based Vectors. *J. Vis. Exp.* **2019**, *143*, e58960.
